# E-cadherin expression as a prognostic factor in patients with ovarian cancer: a meta-analysis

**DOI:** 10.18632/oncotarget.18898

**Published:** 2017-06-30

**Authors:** Chencheng Dai, Jian Cao, Yu Zeng, Sujuan Xu, Xuemei Jia, Pengfei Xu

**Affiliations:** ^1^ Nanjing Maternal and Child Health Institute, Nanjing Maternal and Child Health Care Hospital, Obstetrics and Gynecology Hospital Affiliated to Nanjing Medical University, Nanjing, 210004, China; ^2^ The First Clinical Medical College of Nanjing Medical University, Nanjing, 210029, China; ^3^ Department of Gynecology, Nanjing Maternal and Child Health Care Hospital, Obstetrics and Gynecology Hospital Affiliated to Nanjing Medical University, Nanjing, 210004, China; ^4^ Department of Clinical Laboratory, Nanjing Maternal and Child Health Care Hospital, Obstetrics and Gynecology Hospital Affiliated to Nanjing Medical University, Nanjing, 210004, China

**Keywords:** E-cadherin, ovarian cancer, prognosis, meta-analysis, hazard ratio

## Abstract

The prognostic role of epithelial cadherin (E-cadherin) downregulation in ovarian cancer has been assessed for years while the results remain inconclusive. The aim of our study was to assess this issue. Eligible studies were identified through searches of PubMed, EMBASE and Cochrane Database. In total, 1562 patients from 17 studies were included to assess the association between E-cadherin expression and overall survival/progression-free survival and clinicopathological characteristics of ovarian cancer patients. Hazard ratios (HRs) or odds ratios (ORs) with 95% confidence interval (95% CI) were calculated to estimate the effect. The quality of 17 studies was evaluated using the Newcastle Ottawa Quality Assessment Scale. We also performed subgroup analysis, publication bias and sensitivity analysis in this meta-analysis. The results showed that negative E-cadherin expression significantly predicted poor overall survival of ovarian cancer patients (HR = 1.90, 95% CI = 1.50–2.40). However, negative E-cadherin was not associated with poor progression-free survival (HR = 1.19, 95% CI = 0.86–1.64). Moreover, Negative E-cadherin expression was distinctly associated with FIGO stage (OR = 0.42, 95% CI = 0.31–0.57), tumor grade (OR = 0.48, 95% CI = 0.34–0.67), metastasis (OR = 0.13, 95% CI = 0.07–0.26) and recurrence (OR = 0.48, 95% CI = 0.29–0.79). This meta-analysis revealed that negative E-cadherin expression might be a predicative factor of poor prognosis in ovarian cancer patients.

## INTRODUCTION

Ovarian cancer (OC) is the leading cause of death among gynecologic malignant tumors and significantly impacts the life and health of women worldwide [1]. According to the latest American cancer statistics, approximately 22,280 cases of ovarian cancer are expected to be diagnosed in 2016, and it is estimated that 14,240 women will die from ovarian cancer, representing 5% of all cancer deaths among American women [2]. Even with modern standard treatment for advanced ovarian cancer, the majority of ovarian cancer patients will develop recurrence within 18 months [3]. Thus, the prognosis of ovarian cancer is very poor. CA125 [4], HE4 [5] and several clinicopathological features, including International Federation of Gynecology and Obstetrics stage (FIGO), tumor grade, and distant metastasis, have been recognized as prognostic factors for patients with ovarian cancer [6, 7]. However, due to the biological complexity of ovarian cancer, patients with similarly staged ovarian cancer as well as those treated with the same therapies may exhibit significantly different outcomes. Thus, the currently used clinical prognostic factors are insufficient to accurately predict an individual patient's prognosis. Therefore, it is necessary to identify better prognostic factors to offer timely and effective treatments for OC patients.

Epithelial cadherin (E-cadherin, also known as cadherin 1) is a transmembrane glycoprotein that is a member of calcium adhesion protein molecule family [8]. E-cadherin localizes to cell-cell borders and is strongly expressed in adherens junctions to maintain the integrity and polarity of epithelial cells [9]. E-cadherin plays an important role in the invasion and metastasis of a variety of cancers[10]. The loss or downregulation of E-cadherin expression can lead to several pathological changes: loss of contact inhibition, uncontrolled growth and tumor cell dedifferentiation [11, 12]. In the absence of E-cadherin, the connections between cells become loose and disorganized, thereby promoting invasion.

Many research studies have focused on the expression of E-cadherin and its influence on ovarian cancer prognosis. To date, many studies have confirmed that negative E-cadherin expression in ovarian cancer cells is correlated with advanced tumor progression and predicts poor prognosis [13–16]. In 2012, Peng, H. L. et al. conducted a meta-analysis focused on nine published studies and concluded that negative E-cadherin expression may be associated with a lower overall survival rate in 915 ovarian cancer patients [17]. However, after 2012, many further studies have explored the association of E-cadherin expression and the prognosis of OC patients and have reached different conclusions. Huang, H.N. et al. suggested that among ovarian clear cell carcinoma patients, the negative expression of E-cadherin is a prognostic marker only in the context of activation of the PI3K-Akt pathway [18]. Liew, P. L. et al. suggested that no relationship exists between negative E-cadherin expression and the survival rate of patients with ovarian cancer [19]. Thus, it remains difficult to determine whether negative E-cadherin expression is an independent negative prognostic factor. In addition, it remains unclear whether the differences in these studies are due to small sample sizes or other factors. Therefore, we conducted this meta-analysis with 1562 patients from 17 studies [13–16, 18–30] to investigate the relationship between E-cadherin expression and the clinicopathological features and survival of OC patients. Our result will help provide evidence for effective strategies for the further treatment of ovarian cancer.

## RESULTS

### Selected studies

**Figure 1:**
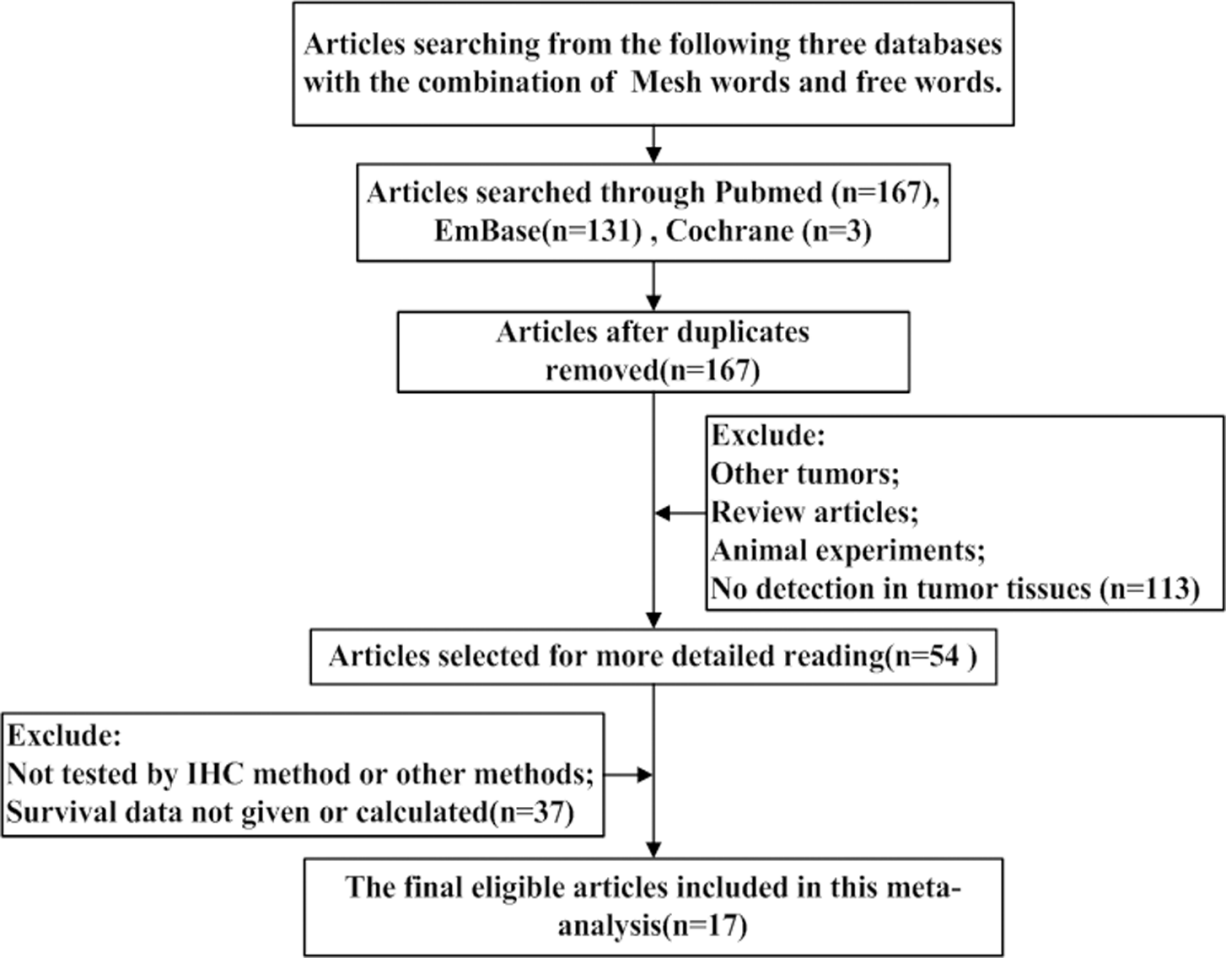
The flow diagram of studies selection

We conducted a search for articles discussing E-cadherin expression and OC patient prognosis, and 167 articles were considered relevant after removal of duplicates. Among these articles, 150 articles were excluded for the following reasons: 1) the study involved non-ovarian tumors; 2) the study did not involve human patients; 3) the article was a review article; 4) E-cadherin expression was not tested using immunohistochemistry (IHC) or other methods; and 5) the hazard ratio (HR) and 95% confidence interval (CI) could not be obtained or calculated according to the information contained in the article. In total, 17 studies were considered eligible. The flow diagram of the selection process is depicted in Figure 1.

### Study characteristics

Seventeen studies were selected for our meta-analysis, and the details of these studies are presented in Table 1. These studies were published between 1996 and 2017. E-cadherin expression was verified by IHC in all studies. The sample size of each article was ranged from 20 to 282 patients. A total of 1562 patients from China, Korea, Japan, Germany, Finland, Croatia, Turkey and France were included in the studies, and HRs and 95% CIs were evaluated. The study follow-up periods ranged from 1 to 334 months.

**Table 1:** Main characteristics and results of the eligible studies

NO	First author	Year	Study location	No. of patients	hystopathological subtypes	Cutoff value	Antibody	Survival	Methods of HR estimation	Pool HR and 95% CI	
										OS	PFS
1	Darai, E.	1996	France	20	10 serous and 10 mucinous	10%	R & D Systems	OS	Survival curves	1.22 (1.01, 1.42)	/
2	Faleiro Rodrigues, C.	2004	Portugal	104	56 serous, 22 mucinous, 16 clear cell, 8 endometrioid and 2 transitional cell	0%	Transduction Laboratories, USA	OS	Given by author	4.83 (1.38, 16.9)	/
3	Voutilainen, K. A.	2006	Finland	282	102 serous, 30 mucinous, 74 endometrioid, 30 clear cell, 46 miscellaneous	5%	Zymed Laboratories, USA	OS	Survival curves	1.70 (0.71, 2.69)	/
4	Cho, E. Y.	2006	Korea	95	95 serous	10%	DiNonA, Seoul, Korea	OS	Survival curves	1.23 (1.12, 1.80)	/
5	Blechschmidt, K.	2008	Germany	48	primary serous	10%	Transduction Laboratories, USA	OS	Given by author	2.82 (1.30, 6.30)	/
6	Shim, H. S.	2009	Korea	72	72 serous	25%	DAKO, Denmark	OS	Survival curves	1.82 (1.32, 2.86)	/
7	Ho, C. M.	2010	Taipei, China	58	58 Clear Cell	10%	DAKO, Denmark	OS, PFS	Given by author	2.30 (1.10, 4.81)	1.45 (0.75, 2.95)
8	Dian, D.	2011	Germany	100	100 serous	25%	DAKO, Denmark	OS, PFS	Survival curves	1.68 (0.51, 2.85)	1.81 (0.80, 4.10)
9	Huang, K. J.	2012	China	136	No given	5%	Santa Cruz, CA	OS	Given by author	1.15 (0.63, 2.09)	/
10	Taskin S.	2012	Turkey	30	30 serous	Product > 3 points	Zymed Laboratories, USA	OS	Given by author	9.60 (2.10, 43.60)	/
11	Bacic, B.	2013	Croatia	54	54 serous	10%	DAKO, Denmark	OS	Given by author	3.08 (1.54, 6.18)	/
12	Huang, H. N.	2014	Taipei, China	72	72 clear cell	10%	DAKO, Denmark	OS, PFS	Given by author	0.70 (0.31, 1.58)	0.71 (0.32, 1.56)
13	Wang, Y.	2014	China	54	No given	10%	Santa Cruz, CA	OS	Given by author	2.92 (1.52, 3.24)	/
14	Mise, B. P.	2015	Croatia	98	98 Serous	10%	DAKO, Denmark	OS, PFS	Given by author	2.70 (1.30, 5.90)	1.35 (0.70, 2.70)
15	Liew, P. L.	2015	Taipei, China	108	47 serous, 23 mucinous, 13 endometrioid and 25 clear cell	Not given	DAKO, Denmark	OS, PFS	Given by author	1.15 (0.58, 2.31)	0.92 (0.46, 1.85)
16	Yu, L.	2015	China	150	114 serous, 21 mucinous, 9 endometrioid and 6 clear cell	Product = 2 points	Maixin, Fuzhou, China	OS	Survival curves	1.99 (1.06, 3.74)	/
17	Sundov, D	2017	Croatia	81	81 serous	10%	DAKO, Denmark	OS	Survival curves	3.30 (1.90–5.80)	/

### Quality assessment

The number of stars on the Newcastle Ottawa Quality Assessment Scale (NOS) for each study ranged from 5 to 9. The average number of NOS stars was 7.41. Sixteen studies were designated as high-quality (NOS stars ≥ 6). Only one study was identified as low-quality (NOS stars < 6). The details of the quality evaluation for each enrolled study are presented in Supplementary Table 1.

### Overall survival and progression-free survival

**Figure 2:**
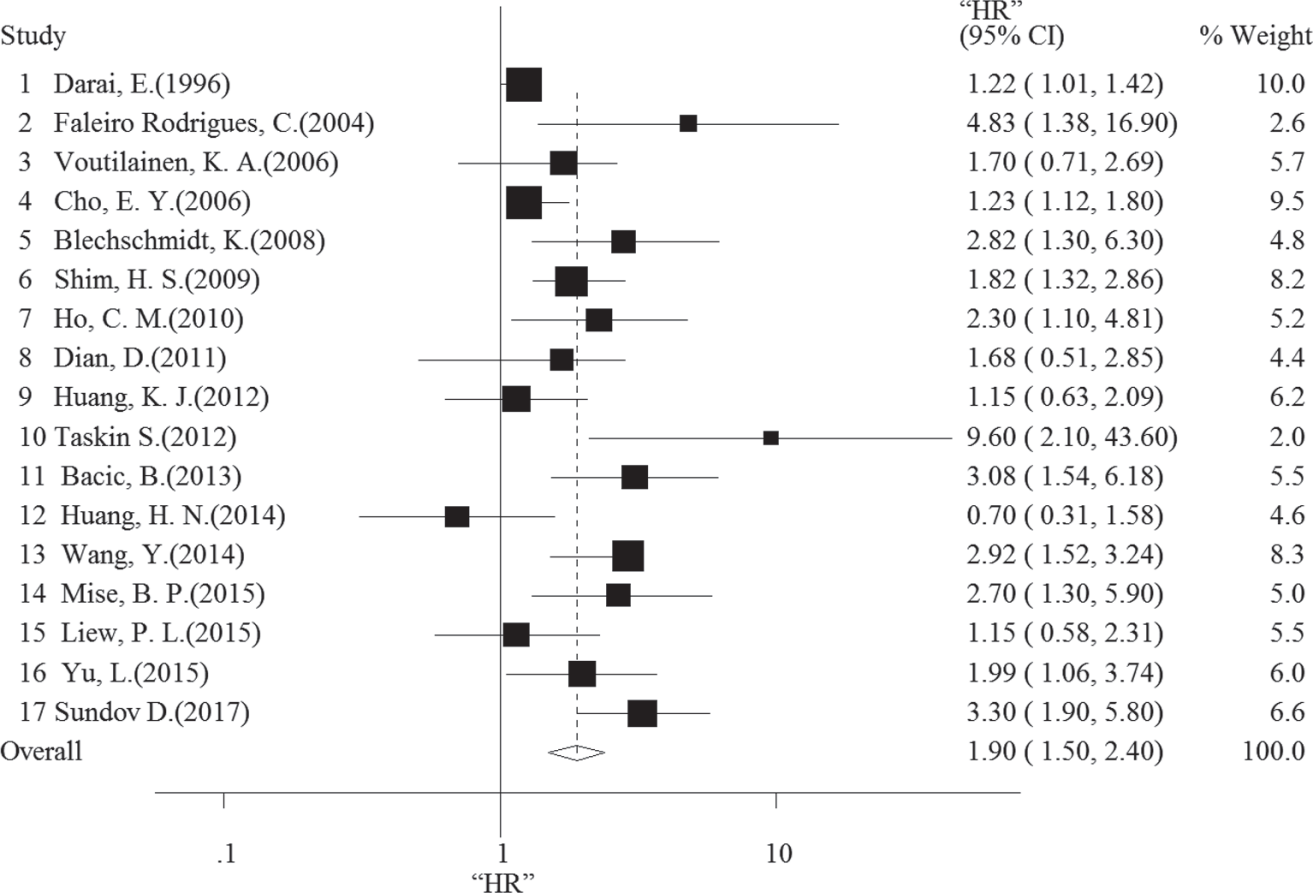
Forest plot shows that negative E-cadherin expression indicates a poor OS of patients with ovarian cancer

First, we conducted an analysis of negative E-cadherin expression and overall survival (OS) in OC patients based on the results of the 17 studies. The random-effects model was chosen because of heterogeneity (I^2^ = 67.5%, *p* < 0.001). As shown in Figure 2, the combined HR was 1.81 (95% CI = 1.44–2.29, *p* < 0.001), which indicates that negative E-cadherin expression was associated with poor OS. However, the combined HR for the 5 studies evaluating a relationship between E-cadherin expression and progression-free survival (PFS) was 1.19 (95% CI = 0.86–1.64, *p* = 0.459, fixed-effects model) without heterogeneity (I^2^ < 50%, *p* = 0.302) (Supplementary Figure 1). This result suggests that negative E-cadherin expression did not indicate poor PFS among OC patients.

### Subgroup analysis

We performed subgroup analyses for the number of patients, publication year, cut-off value, antibody source and study country. The results of each subgroup consistently indicated that negative E-cadherin expression is correlated with poor prognosis. The details of the subgroup analysis are presented in Supplementary Table 2.

### The correlation between E-cadherin expression and clinicopathological features

We selected clinical features that were presented in more than 3 articles to conduct a correlation analysis between E-cadherin expression and clinical parameters. As shown in Table 2, there was no correlation between E-cadherin expression and the following three clinical parameters: patient age, histologic type, and lymph node or vascular invasion. However, negative E-cadherin expression was correlated with FIGO stage (I/II vs. III/IV, pooled OR = 0.42, 95% CI = 0.31–0.57, *p* < 0.001), tumor grade (1/2 vs. 3, pooled OR = 0.48, 95% CI = 0.34–0.67, *p* < 0.001), metastasis (Absent vs. Present, pooled OR = 0.13, 95% CI = 0.07–0.26, *p* < 0.001) and recurrence (Absent vs. Present, pooled OR = 0.48, 95% CI = 0.29–0.79, *p* < 0.001).

**Table 2:** Main results for meta-analysis between negative E-cadherin expression and clinicopathological features

Association between E-cadherin and clinical features	No. Reference Studies	Overall OR (95% CI)	Heterogeneity test (Q, I^2^, p)
Age (< 50 vs. ≥ 50)	Shim, H. S. [23], Huang, H. N. [18], Wang, Y. [27]	0.94 (0.56,1.65)	6.75, 70.4%, 0.83 (random-effected)
FIGO stage (I/II vs. III/IV)	Darai, E. [20], Faleiro Rodrigues, C. [13], Voutilainen, K. A. [21], Cho, E. Y. [22], Shim, H. S. [23], Dian, D. [24], Huang, H. N. [18], Wang, Y. [27], Yu, L. [29]	**0.42 (0.31,0.57)**	39.97, 80.0%, < 0.01 (random-effected)
Tumor grade (1/2 vs. 3)	Darai, E. [20], Faleiro Rodrigues C. [13], Voutilainen, K. A. [21], Cho, E. Y. [22], Shim, H. S. [23], Dian, D. [24], Bacic, B. [26], Wang, Y. [27]	**0.48 (0.34,0.67)**	9.17, 23.7%, < 0.01 (fixed-effected)
Histologic type (serous vs. others)	Darai, E. [20], Faleiro Rodrigues, C. [13], Voutilainen, K. A. [21], Yu, L. [29]	1.43 (0.93,2.19)	2.02, 0.0%, < 0.01 (fixed-effected)
Metastasis (Absent vs. Present)	Cho, E. Y. [22], Dian, D. [24], Wang, Y. [27], Yu, L. [29]	**0.13 (0.07,0.26)**	5.04, 40.5%, < 0.01 (fixed-effected)
Lymph node or vascular invasion (Absent vs. Present)	Faleiro Rodrigues, C. [13], Dian, D. [24], Bacic, B. [26], Huang, H. N. [18]	0.67 (0.36,1.24)	3.80, 21.0%, 0.20 (fixed-effected)
Recurrence (Absent vs. Present)	Darai, E. [20], Voutilainen, K. A. [21], Cho, E. Y. [22], Shim, H. S. [23]	**0.48 (0.29,0.79)**	1.25, 0.0%, < 0.01 (fixed-effected)

### Sensitivity analysis

The sensitivity of the pooled data from the 17 studies was evaluated. As shown in Table 3, no significant change was observed in the corresponding pooled HRs, indicating that our conclusion that negative E-cadherin expression predicts poor OS among OC patients is reliable.

**Table 3:** Results of the sensitivity analysis

Excluded study	HR	95% CI
1 Darai, E. (1996)	2.00	1.55–2.57
2 Faleiro Rodrigues, C. (2004)	1.85	1.46–2.33
3 Voutilainen, K. A. (2006)	1.91	1.50–2.45
4 Cho, E. Y. (2006)	2.00	1.54–2.60
5 Blechschmidt, K. (2008)	1.86	1.46–2.36
6 Shim, H. S. (2009)	1.91	1.48–2.47
7 Ho, C. M. (2010)	1.88	1.47–2.40
8 Dian, D. (2011)	1.91	1.50–2.44
9 Huang, K. J. (2012)	1.96	1.54–2.51
10 Taskin S. (2012)	1.83	1.46–2.30
11 Bacic, B. (2013)	1.84	1.45–2.34
12 Huang, H. N. (2014)	1.99	1.57–2.52
13 Wang, Y. (2014)	1.80	1.43–2.27
14 Mise, B. P. (2015)	1.86	1.46–2.37
15 Liew, P. L. (2015)	1.96	1.53–2.50
16 Yu, L. (2015)	1.89	1.48–2.42
17 Sundov D. (2017)	1.81	1.44–2.29

### Publication bias

**Figure 3:**
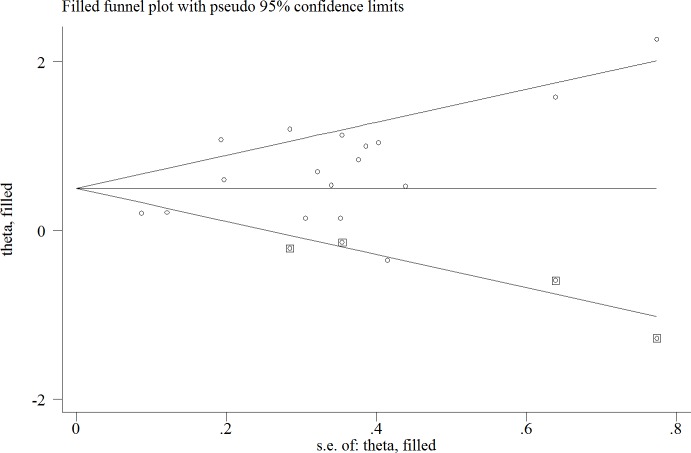
Trim-and-fill analysis estimating the number missing studies for the association between negative E-cadherin and survival outcomes of patients with ovarian cancer The squares represent the possible missing studies.

Both Begg's and Egger's funnel plot were generated to assess the publication bias. The distribution of Egger's funnel plot appeared asymmetrical in the heterozygote comparison (*p* = 0.016) (Supplementary Figure 2). Thus, we used a trim-and-fill method to adapt the publication bias. First, three articles with smaller numbers of patients were excluded to generate a symmetrical plot. We considered only the larger studies to estimate an adjusted summary effect. Subsequently, we replicated the funnel plot to replace the excluded studies with their ‘missing’ counterparts around the adjusted summary estimate. Finally, we applied the random-effects model to obtain the adjusted estimate: HR of 1.64 (1.31–2.06) for negative E-cadherin expression (Figure 3). Whether we applied the trim-and-fill method or not, the meta-analysis did not produce conflicting conclusions, suggesting that our results were statistically stable.

## DISCUSSION

In 2012, the meta-analysis by Peng, H. L. et al. reported that negative E-cadherin expression predicts worse OS in OC patients [17], However, not all the related studies reported consistent conclusions. In 2014, Huang, H. N. et al. suggested that E-cadherin expression had no impact on prognosis in OC patients if the PI3K-Akt pathway was not activated [18]. Liew, P. L. et al. reported that negative E-cadherin expression did not predict poor prognosis among patients with ovarian cancer. In addition, additional papers published after 2012 continued to explore the relationship between E-cadherin expression and the prognosis of OC patients [16, 18, 19, 26–30]. Therefore, we conducted this meta-analysis to verify whether negative E-cadherin expression is an independent prognosis factor for OC patients.

In our study, we included additional studies to the subset examined by Peng, H.L. et al. to evaluate the prognostic value of negative E-cadherin expression in OC patients. Finally, we comprehensively conducted this meta-analysis involving 1562 patients from 17 independent studies to explore the association between negative E-cadherin expression and OS or PFS among OC patients. Our results indicate that the negative expression of E-cadherin is correlated with a significant reduction in OS (HR: 1.90 (1.50, 2.40)) but not PFS among OC patients (HR: 1.19 (0.86, 1.64)). Furthermore, we also tested the quality of the 17 papers included in our analysis using NOS, and a trim-and-fill test was used to detect publication bias of all studies. Sensitivity analysis was used to observe the reliability of the results. To the best of our knowledge, we have conducted the largest and most comprehensive meta-analysis of the association between negative E-cadherin expression and the survival of OC patients.

We also performed a comprehensive analysis to investigate the relationship between negative E-cadherin expression and clinicopathological features. The results revealed that negative E-cadherin is associated with certain clinicopathological features, including FIGO stage, tumor grade, metastasis and recurrence. Moreover, previous studies reported that FIGO stage, tumor grade and recurrence may be considered independent prognostic factors for OC patients [31, 32]. Therefore, our results suggest that negative E-cadherin expression might be a more effective predictor of poor prognosis in OC patients.

According to our results, negative E-cadherin expression predicts poor OS (HR: 1.90 (1.50, 2.40)) in OC patients. OS is considered the “gold standard” in oncology trials because of its relevance and objectivity [33]. However, an increasing number of studies recognize that confounding effects of post-study therapies and trial crossover are often present in clinical trials that rely on OS, and PFS is, therefore, the most commonly surrogate endpoint in oncology trials [33]. However, negative E-cadherin expression was not correlated with the PFS of OC patients (HR: 1.19 (0.86, 1.64)). The inability to detect a relationship between these two factors may be the result of the limited numbers of samples (*n* = 436). Thus, more studies were needed to accurately evaluate the true association between E-cadherin expression and PFS in OC patients.

Previous studies have reported that negative expression of E-cadherin is closely related to cell-cell adhesion [34] and the mobility and proliferation [35] of epithelial cells. In specific physiological and pathological conditions, the loss of E-cadherin expression in ovarian epithelial cells promotes loss of cellular polarity and cellular adhesion and enhances migration and movement. Moreover, E-cadherin expression is negatively correlated with dedifferentiation and lymph node metastasis [36, 37]. In addition, E-cadherin is a downstream target of the Wnt [38] and PI3K-AKT pathways [39]. Thus, negative E-cadherin expression is associated with tumor progression. According to our meta-analysis, negative E-cadherin expression in OC is correlated with FIGO stage (I/II vs. III/IV, pooled OR = 0.42, 95% CI = 0.31–0.57, *p* < 0.001), tumor grade (1/2 vs. 3, pooled OR = 0.48, 95% CI = 0.34–0.67, *p* < 0.001), metastasis (Absent vs. Present, pooled OR = 0.13, 95% CI = 0.07–0.26, *p* < 0.001), and with ovarian cancer recurrence (Absent vs. Present, pooled OR = 0.48, 95% CI = 0.29–0.79, *p* < 0.001). These results indicate that E-cadherin may serve as an independent prognostic factor.

There are some limitations that should be acknowledged in this meta-analysis. First, the search strategy was restricted to three databases (PubMed, EMBASE and Cochrane) and to articles published in English or Chinese, which may have led to the exclusion of some relevant studies. Second, the different cut-off values for negative E-cadherin expression in these studies may have resulted in inaccurate results and inconsistent conclusions. Third, the use of different E-cadherin antibodies may have produced different results. In addition, several HRs and 95% CIs for the relationship between E-cadherin expression and PFS were not provided. Moreover, the clinicopathological features of many studies were not reported. Therefore, biases were unavoidable in this meta-analysis. Further evidence is required to assess the association between negative E-cadherin expression and PFS in OC patients. Despite these limitations, we have high confidence based on the results of this meta-analysis that negative E-cadherin expression is significantly associated with OS among OC patients.

In conclusion, our study demonstrates that negative E-cadherin expression is associated with worse prognosis among patients with ovarian cancer. In addition, clinicopathological features, such as FIGO stage, tumor grade, metastasis and recurrence, are significantly associated with negative E-cadherin expression. Based on our studies, an evaluation of E-cadherin expression may provide relatively accurate OS prognostic information among OC patients. Therefore, our study promotes effective strategies for the further treatment of ovarian cancer.

## MATERIALS AND METHODS

### Search strategy

We searched for all relevant studies on E-cadherin expression and ovarian cancer prognosis using the PubMed, EMBASE and Cochrane databases. We used the following term combinations for our search: “ovarian neoplasm or ovarian cancer or OC or ovarian carcinoma or ovarian tumor”, “Epithelial cadherin or cadherin 1 or E-cadherin” and “prognosis or prognoses or survival outcome”. The last search was updated on February 1, 2017. The reference lists of reviews and primary studies were also searched, and the study authors were approached for help if necessary.

### Data extraction

First, we extracted the following information from the included articles: the first author's name, publication year, study country, number of patients, cut-off value, antibody source, age, FIGO stage, tumor grade, histological type, presence of lymph node or vascular invasion, metastasis, recurrence, HR, 95% CI and E-cadherin expression-related survival. If the HR and 95% CI were not provided, we calculated these values from the Kaplan-Meier curve using the method reported by Chaimani, A. et al. [40]. These calculations were performed independently by two researchers. When the calculations disagreed, mistakes were identified and corrected after discussion.

### Methodological quality assessment

The NOS is the recommended tool to assess the quality of cohort and case-control studies [41, 42]. In the present work, two independent researchers adopted the NOS to assess the quality of the 17 included studies. The NOS is composed of three major parts: Selection, Comparability and Outcome. Each section also includes several detailed entries. The NOS adopts a semi-quantitative scoring system for assessment. The entry entitled ‘Selection’ and ‘Outcome’ can earn a maximum of one stars, whereas the entry entitled ‘Comparability’ can earn a maximum of two stars. The total number of possible starts is nine. Higher star counts reflect higher-quality studies. Studies ≥ 6 stars according to the NOS method are considered high quality, and studies with < 6 stars are considered low quality.

### Statistical analysis

HRs and 95% CIs were used to evaluate the relationship between negative E-cadherin expression and OC patient prognosis. Heterogeneity among the studies was assessed using the *Q* test and I^2^ index. Heterogeneity was present when *p* < 0.10 and I^2^ > 50%, and the random-effects model was utilized. Otherwise, we adopted the fixed-effect model. If the combined HR and 95% CI were greater than 1.0, the relationship between negative E-cadherin expression and OS or PFS was considered statistically significant. To investigate the cause of high heterogeneity, we performed subgroup analysis. All 17 studies were divided into several groups according to the number of patients, publication year, cut-off value, antibody source and study location. Subsequently, the HRs, 95% CIs and I^2^ indexes of the different groups were calculated [43]. The association between negative E-cadherin expression and clinical pathologic features such as patient age, FIGO stage, tumor grade, histologic type, metastasis, the presence of lymph node or vascular invasion, and recurrence was evaluated using the ORs and 95% CIs. If the OR and 95% CI did not overlap 1.0, negative E-cadherin expression was considered to be statistically correlated with the clinical feature. Publication bias was assessed using the Begg's and Egger's tests. *p* < 0.05 was considered to reflect the presence of publication bias. Funnel plots were used to describe the potential publication bias, and a symmetric plot suggested no publication bias. All of the statistical analyses were performed using STATA 10.0 (StataCorp, College Station, TX).

## SUPPLEMENTARY FIGURES AND TABLES


