# Supplementary material for: E-cadherin expression as a prognostic factor in patients with ovarian cancer: a meta-analysis
Source: Oncotarget. 2017 Jun 30;8(46):81052–61. doi: 10.18632/oncotarget.18898 (PMC5655261; doi:10.18632/oncotarget.18898)
Supplement: Supplementary file 1 [file oncotarget-08-81052-s001.pdf]

E-cadherin expression as a prognostic factor in patients with ovarian cancer: a meta-analysis

Supplementary Materials

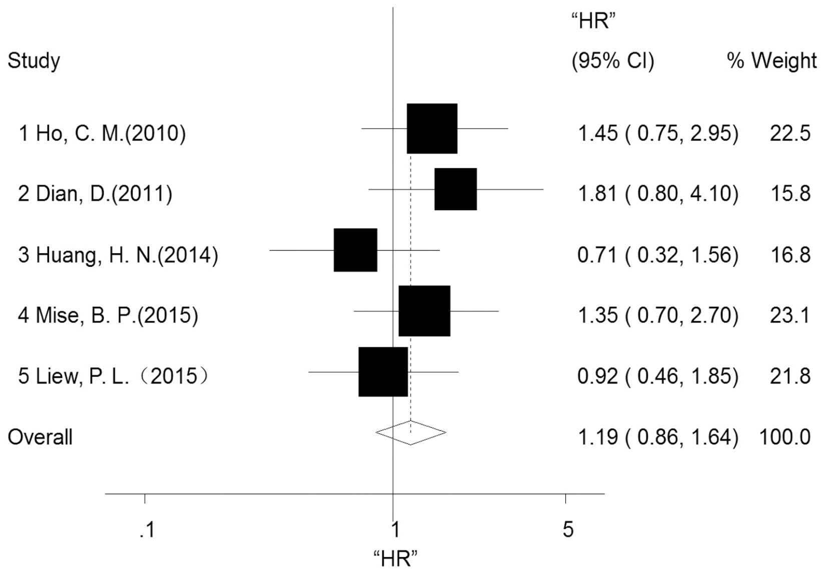

Supplementary Figure 1: Forest plot shows the relation of negative E-cadherin expression with PFS of patients with ovarian cancer.

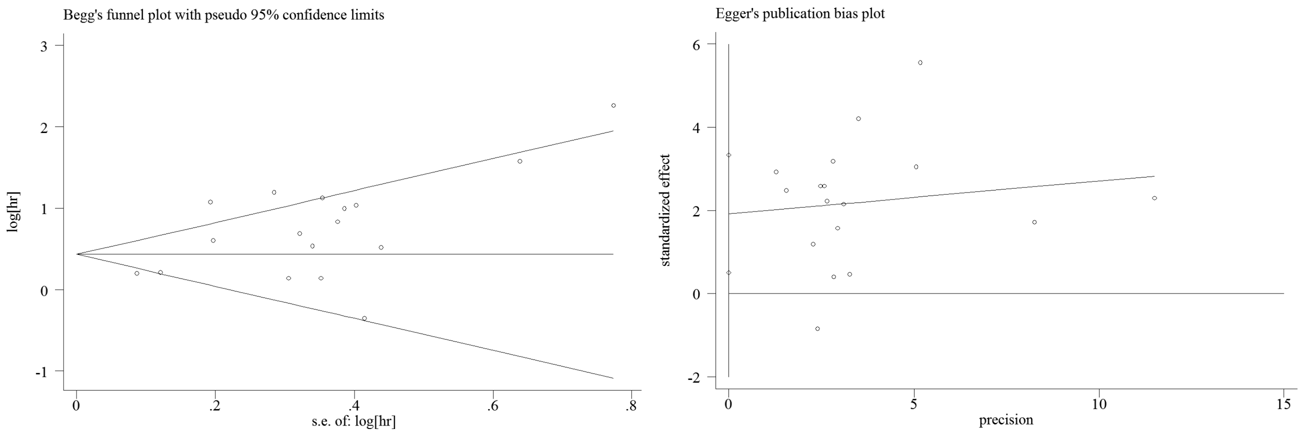

Supplementary Figure 2: Begg's and Egger's funnel plot evaluated the publication bias of the selected studies.

Supplementary Table 1: Quality assessment of studies (for cohort studies)

Studyid	Selection				Comparability	Outcome			Total Points
	Representativeness of the Exposed Cohort	Selection of the Non-Exposed Cohort	Ascertainment of Exposure	Demonstration That Outcome of Interest Was Not Present at Start of Study	Comparability of Cohorts on the Basis of the Design or Analysis	Assessment of Outcome	Was Follow-Up Long Enough for Outcomes to Occur	Adequacy of Follow Up of Cohorts	
1 Darai, E. (1996)	*	*	*	*	**		*		7*
2 Faleiro Rodrigues, C. (2004)	*	*	*	*	*	*	*	*	8*
3 Voutilainen, K. A. (2006)	*	*	*	*	*	*	*	*	8*
4 Cho, E. Y. (2006)	*	*	*	*	*	*	*	*	8*
5 Blechschmidt, K. (2008)	*	*	*	*	*	*	*	*	8*
6 Shim, H. S. (2009)	*	*	*	*	**	*	*		8*
7 Ho, C. M. (2010)	*	*	*	*		*	*		6*
8 Dian, D. (2011)	*	*	*	*	**	*	*	*	9*
9 Huang, K. J. (2012)	*	*	*	*			*		5*
10 Taskin S. (2012)	*	*	*			*	*	*	6*
11 Bacic, B. (2013)	*	*	*	*	*	*	*	*	8*
12 Huang, H. N. (2014)	*	*	*	*	**	*	*	*	9*
13 Wang, Y. (2014)	*	*	*	*	**		*		7*
14 Mise, B. P. (2015)	*	*	*	*	**	*	*	*	9*
15 Liew, P. L. (2015)	*	*	*	*		*	*		6*
16 Yu, L. (2015)	*	*	*	*	**	*	*		8*
17 Sundov D. (2017)	*	*	*	*		*	*		6*

Supplementary Table 2: Subgroup analysis of negative E-cadherin and prognosis of ovarian cancer patients

Stratified Analysis	No. of Study	Pools HR (95% CI)	*p*-value	I^2	Heterogeneity *p*-value
**No. of Patients**					
≤ 90	9	2.19 (1.49,3.22)	< 0.001	80.9%	< 0.001
> 90	8	1.41 (1.18,1.69)	< 0.001	31.4%	0.177
**Years**					
1996−2011	8	1.59 (1.26, 2.00)	< 0.001	50.9%	0.047
2012−2017	9	2.08 (1.42,3.07)	< 0.001	67.4%	0.002
**Cut-off Value**					
10%	9	1.96 (1.40,2.74)	< 0.001	80.7%	< 0.001
Others	8	1.73 (1.38,2.18)	< 0.001	35.6%	0.144
**Antibody**					
DAKO, Denmark	8	1.94 (1.39,2.70)	< 0.001	51.8%	0.042
Others	9	1.87 (1.36,2.57)	< 0.001	75.7%	< 0.001
**Study Location**					
Europe	9	2.49 (1.62,3.82)	< 0.001	74.6%	< 0.001
Asia	8	1.57 (1.15, 2.15)	0.004	68.3%	0.002
